# *Purpureocillium lilacinum* Keratitis Outbreak Associated with an Ophthalmology Clinic — New York City, 2024

**DOI:** 10.15585/mmwr.mm7505a1

**Published:** 2026-02-12

**Authors:** Michelle E. Chang, Elise Mantell, William G. Greendyke, Brian Schwem, Zachary Mudge, Luisa F. López, Dallas J. Smith, Anastasia P. Litvintseva, Adam Rowh, Heather Moulton-Meissner, Paige Gable, Tristan D. McPherson

**Affiliations:** ^1^Epidemic Intelligence Service, CDC; ^2^New York City Department of Health and Mental Hygiene, New York, NY; ^3^Division of Foodborne, Waterborne, and Environmental Diseases, National Center for Emerging and Zoonotic Infectious Diseases, CDC; ^4^Division of Healthcare Quality Promotion, National Center for Emerging and Zoonotic Infectious Diseases, CDC.

**Summary**

**What is already known about this topic?**

*Purpureocillium lilacinum* is an environmental mold that can cause a range of infections in humans. Although infection in immunocompetent persons is uncommon, eye infections have been reported.

**What is added by this report?**

Three patients developed *P. lilacinum* corneal infections after laser eye surgery at a New York City ophthalmology clinic, one of whom required corneal transplantation. Investigation identified infection prevention and control deficiencies at the clinic that potentially resulted in mold exposure. After implementation of infection prevention and control measures, no further cases were identified.

**What are the implications for public health practice?**

Adherence to published infection prevention and control guidance during all procedures in ambulatory settings can reduce the risk for health care–associated infections and related complications.

## Abstract

In December 2024, a clinical laboratory notified the New York City Health Department of three patients with fungal keratitis caused by an unidentified mold following laser eye surgery at an ophthalmology clinic. All three patients experienced vision loss, and one patient required corneal transplantation. Corneal cultures from two of the patients grew *Purpureocillium lilacinum*, an environmental mold that can cause a range of infections in humans; eye infections have been reported. The clinic paused surgeries. The health department assessed the clinic’s infection prevention and control (IPC) practices and identified multiple deficiencies, including incomplete instrument sterilization logs, absence of disinfectants approved for use on work surfaces, use of expired topical ocular medications, and opportunities for exposure to nonsterile water from cool-mist humidifiers in the procedure room. CDC environmental infection control guidelines recommend avoidance of cool-mist humidifiers in health care facilities. All environmental cultures were negative for *P. lilacinum*, but fungal amplicon sequencing detected *P. lilacinum* DNA in the tubing of a surgical device. No further cases were identified after implementation of recommended IPC measures and resumption of surgeries during January 2025. Adherence to published IPC guidance during all procedures in ambulatory settings can reduce the risk for health care–associated infections.

## Introduction

In December 2024, a clinical laboratory in New York City (NYC) notified the NYC Health Department of three patients with fungal keratitis caused by an unidentified mold that developed after elective laser eye surgery performed at an ophthalmology clinic for the purpose of reducing or eliminating the need for corrective lenses. The clinic had suspected infection based on patient reports of eye pain and vision loss and postoperative clinical assessments, and sent corneal specimens to the laboratory, which noted fungal elements on microscopy. Because of the unusual appearance of the organism, the laboratory notified the health department. Corneal cultures from two of the patients grew *Purpureocillium lilacinum* (formerly *Paecilomyces lilacinus*), an environmental mold. Infection with *P. lilacinum* can result in clinical manifestations ranging from superficial mycoses to life-threatening systemic infections (*1*); eye infections have been reported (*2*,*3*). *P. lilacinum* infection is not reportable in NYC.

## Investigation and Findings

### Initial Clinic Response and Laboratory Report to Health Department

**FIGURE 1:**
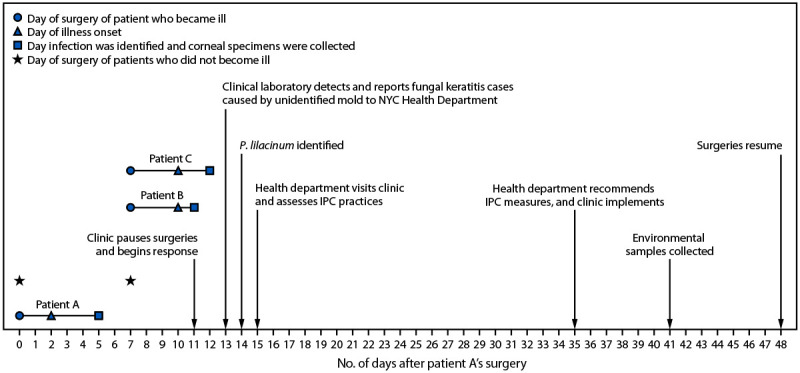
Timeline for *Purpureocillium lilacinum* keratitis outbreak — New York City, December 2024–January 2025* **Abbreviations:** IPC = infection prevention and control; NYC = New York City. * Of the eight patients who did not become ill, two had surgery on day 0, and six had surgery on day 7. The analysis period lasted from days 0–13.

**FIGURE 2:**
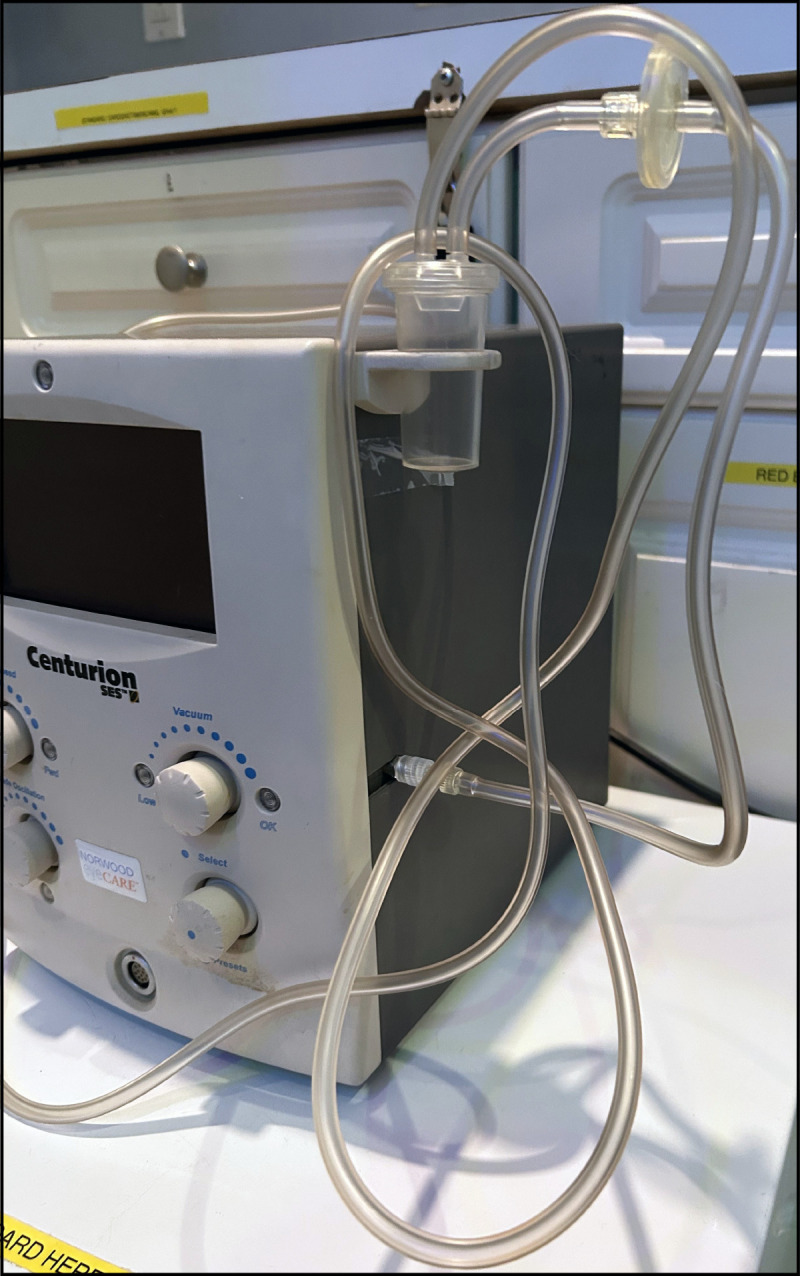
Epikeratome machine and single-use tubing from the ophthalmology clinic — New York City, December 2024* Photo/Michelle Chang. * An epikeratome is a surgical device that uses suction to separate the corneal epithelium from the underlying corneal layers. The epikeratome consists of the machine that generates suction, suction tubing, and a handheld attachment that touches the eye during surgery and is sterilized after each use. The handheld attachment is not included in the photo.

The clinic employed one ophthalmologist and consisted of a single treatment room. Patient A had surgery on day 0 and experienced symptoms 2 days later (Figure 1). Patients B and C had surgery on day 7 and became symptomatic 3 days later, on day 10. After identifying infections in patients A and B (on days 5 and 11, respectively), the clinic paused surgeries. A third infection, in patient C, was identified the next day (day 12). Clinic staff members conducted a round of environmental cleaning, discarded or replaced some equipment, and retained other equipment for future environmental testing. Retained items included the procedure room refrigerator, and an epikeratome, a surgical device that uses suction to separate the corneal epithelium from the underlying corneal layers. The epikeratome consists of the machine that generates suction, suction tubing (Figure 2), and a handheld attachment that touches the eye during surgery which is sterilized after each use.

On day 13, the laboratory detected mold in the clinical specimens and notified the health department. The following day, the mold in patient A’s culture was identified as *P. lilacinum*; *P. lilacinum* was detected in a second patient’s culture shortly thereafter.

In accordance with standard practice for an outbreak investigation, the NYC Health Department’s institutional review board did not review this activity. This activity was reviewed by CDC, deemed not research, and was conducted consistent with applicable federal law and CDC policy.*

### *P. lilacinum* Keratitis Case Definition and Identification

A case of *P. lilacinum* keratitis was defined by the NYC Health Department as eye pain or vision loss in a patient after laser eye surgery at the clinic on days 0–13^†^ and 1) corneal cultures demonstrating *P. lilacinum* or 2) scrapings identifying fungal elements. Cases were identified through medical record review and clinician interviews. Among 11 patients who had laser eye surgery during the analysis period, three patients, who had surgery on day 0 (patient A) and day 7 (patients B and C), experienced illness meeting the case definition 2–3 days after surgery (Figure 1). Two of these three patients received a positive *P. lilacinum* culture result and one patient’s corneal scrapings identified fungal elements. All three ill patients experienced vision loss, and one of the two who received a positive *P. lilacinum* culture result required corneal transplantation. After identification of the fungal infection, the ill patients were started on topical voriconazole and natamycin. Eventually, all were transitioned to oral posaconazole. Patients sought care from multiple providers, and information on clinical outcomes is not available.

### Clinic Procedures and Patient Characteristics

Based on the limited demographic and clinical information collected by the clinic, the health department reviewed information on the age, sex, and comorbidities of the 11 patients who underwent surgery during the analysis period. Surgeries at the clinic were conducted in a highly protocolized manner. All patients treated during the analysis period were in contact with the same surgical equipment in the same operative environment. The surgical procedures for all three ill patients, and at least four of the eight patients who did not become ill, involved use of the epikeratome. Of the eight patients who did not become ill, two had surgery on day 0 and six had surgery on day 7. Patients received the same perioperative medications, including three types of eye drops (steroid, nonsteroidal anti-inflammatory, and antibiotic). None of the patients was known to be immunocompromised, and no apparent demographic, clinical, or procedural differences were identified between patients who did and did not become ill.

### Infection Prevention and Control Assessment and Findings

On day 15, health department staff members visited the clinic to assess infection prevention and control (IPC) practices. The IPC assessment was conducted using CDC’s Infection Control Assessment and Response tool (*4*), which includes modules that can be selected on the basis of facility-specific concerns or applicability to a specific organism. The assessment focused primarily on the domains of instrument sterilization, environmental cleaning, medication safety, and water exposures.

Multiple IPC deficiencies were identified, including incomplete sterilization logs for surgical instruments, use of a disinfectant that is not approved by the Environmental Protection Agency (EPA) for surface cleaning in the procedure room, intraoperative use of expired topical ocular medications, and opportunities for patient exposure to nonsterile water during surgery. For example, clinic staff members reported that an undated bottle of saline solution was used to provide surgical irrigation for multiple patients over the course of weeks. In addition, among the equipment that clinic staff discarded and replaced as part of their initial response to the infections were multiple cool-mist humidifiers. During the IPC assessment, the new cool-mist humidifiers were present in the procedure room.

### Genomic Analysis and Antifungal Susceptibility Testing of *P. lilacinum* Clinical Isolates

Whole genome sequencing analysis of the two *P. lilacinum* clinical isolates demonstrated that they differed by approximately 55 single nucleotide polymorphisms (SNPs) from each other and by approximately 200,000 SNPs from control isolates. This analysis suggested a common infection source. Antifungal susceptibility testing demonstrated good in vitro activity of azoles and supported resistance to amphotericin B.

### Environmental Testing

On day 41, health department staff members collected the saline bottle, stored epikeratome tubing, and swabs from five refrigerator locations (gasket, interior door, interior bottom, interior upper, and medication compartment) for fungal cultures and amplicon sequencing.^§^ The humidifiers in use when the infections occurred had been discarded before the investigation and were not available for testing.

All environmental cultures were negative for *P. lilacinum*, but fungal amplicon sequencing detected *P. lilacinum* DNA in the epikeratome suction tubing (Figure 2). During surgery, the epikeratome draws air away from the eye through a handheld attachment that touches the eye and is connected to suction tubing that contains a filter to prevent flow of debris into the machine. The handheld attachment is sterilized after each use and was therefore not made available for sampling. The suction tubing is intended for single use and is provided as a sterile product. Clinic staff members reported that tubing was never shared between patients; it is not known whether the set of tubing collected for testing was the one used for any of the infected patients’ procedures. The epikeratome machine is a multiuse item. It was not sent for testing because no clear sampling approach could be identified.

## Public Health Response

Whether the *P. lilacinum* DNA in the epikeratome tubing was the source of infection is unclear because of the way the tubing was used and the possibility of environmental contamination. The humidifiers were another possible source. Cool-mist humidifiers were previously implicated in an outbreak of *Mycobacterium chelonae* eye infections at a laser eye surgery clinic (*5*); however, the humidifiers in use at the time the *P. lilacinum* infections in this outbreak occurred had been replaced and were unavailable for testing. CDC environmental infection control guidelines recommend avoidance of cool-mist humidifiers in health care facilities (*6*), and current health care engineering guidelines specify that steam humidification should be used (*7*).

The clinic worked closely with the health department to implement all recommended IPC measures to prevent potential exposures, including exchange of cool-mist humidifiers for steam humidifiers and, out of an abundance of caution, replacement of the epikeratome. No further cases were identified after implementation of IPC measures and resumption of surgeries during January 2025.

## Discussion

*P. lilacinum* caused severe disease in all three infected patients, including one who required corneal transplantation. Most cases of *P. lilacinum* keratitis have been reported in association with soft contact lens use, eye trauma, eye surgery, and immunosuppression (*2*,*3*). *P. lilacinum* is considered intrinsically resistant to amphotericin B, and treatment with polyene antifungals including amphotericin B and natamycin has been linked to poorer clinical outcomes (*1*,*3*,*8*). A recent analysis of fungal culture results from a major U.S. commercial laboratory found increasing rates of *P. lilacinum* detection during 2019–2025 (*8*). Two *P. lilacinum* strains registered with EPA during 2005 and 2021 are used as agricultural bionematicides (biologic agents used to control plant parasites) in the United States (*9*,*10*). Agricultural use of *P. lilacinum* might increase its presence in the environment, potentially contributing to clinical infections and culture contamination. Because *P. lilacinum* is known to cause drug-resistant keratitis, and rates of detection are increasing, *P. lilacinum* should be considered as a potential cause of infection after eye surgery, even before definitive culture identification.

### Implications for Public Health Practice

This investigation identified IPC deficiencies that could have resulted in mold exposure, although none was conclusively linked to the clinic patients’ infections. The prevalence of the IPC findings from this investigation in ophthalmology clinics remains unclear. Adherence to published IPC guidance during all procedures in ambulatory settings can reduce the risk for health care–associated infections and related complications.

## Footnotes

* 45 C.F.R. part 46.102(l)(2), 21 C.F.R. part 56; 42 U.S.C. Sect. 241(d); 5 U.S.C. Sect. 552a; 44 U.S.C. Sect. 3501 et seq.

† No patients were lost to postoperative follow-up during the period when the infections occurred.

§ Fungal amplicon sequencing used the internal transcribed spacer (ITS) regions ITS1 and ITS2, as well as 28S ribosomal DNA, as targets.
